# The eNOS 894G/T gene polymorphism and its influence on early and long-term mortality after on-pump cardiac surgery

**DOI:** 10.1186/1749-8090-8-199

**Published:** 2013-10-25

**Authors:** José Hinz, Daniel Schöndorf, Christian Bireta, Christina Lipke, Onnen Moerer, Ingo Bergmann, Christoph Herman Wiese, Ashham Mansur, Hanna Schotola, Anton Sabashnikov, Michael Quintel, Friedrich Albert Schoendube, Aron Frederik Popov

**Affiliations:** 1 Department of Anaesthesiology, Emergency and Intensive Care Medicine, University of Göttingen, Robert-Koch-Strasse 40, 37099 Göttingen, Germany; 2 Department of Thoracic Cardiovascular Surgery, University of Göttingen, Robert-Koch Strasse 40, 37099 Göttingen, Germany; 3 Department of Cardiothoracic Transplantation & Mechanical Support, Royal Brompton and Harefield Hospital, Harefield, Hill End Road, UB9 6JH London, United Kingdom

## Abstract

**Background:**

The *eNOS* 894G/T polymorphism (GG, GT, and TT) is associated with cardiovascular mortality and may influence cardiovascular diseases as a genetic risk factor. Moreover, this polymorphism has an impact on intraoperative hemodynamics during cardiac surgery with cardiopulmonary bypass (CPB). In this study, we analyzed the influence of this gene polymorphism on early clinical outcome in patients who underwent cardiac surgery with CPB. Also, we performed a 5-year follow-up, assessing the impact of this polymorphism on long-term mortality.

**Method:**

500 patients who underwent cardiac surgery with CPB between 2006 and 2007 were included in this prospective single centre study. Genotyping for the eNOS gene polymorphism was performed by polymerase chain reaction amplification.

**Results:**

Genotype distribution of 894G/T was: GG 50.2%; GT 42.2%; TT 7.8%. Cardiovascular risk factors were equally distributed between the different genotypes of the eNOS 894G/T polymorphism. No significant difference among the groups was shown regarding Euroscore, SAPS II and APACHE II. Perioperative characteristics were also not affected by the genotypes, except for the consumption of norepinephrine (p = 0.03) and amiodarone (p = 0.01) which was higher in the GT allele carrier. The early postoperative course was quite uniform across the genotypes, except for mean intensive care unit length of stay which was significantly prolonged in GT carriers (p = 0.001). The five-year follow-up was 100% complete and showed no significant differences regarding mortality between the groups.

**Conclusion:**

Our results show that the eNOS 894G /T polymorphism is not associated with early and late clinical outcome after cardiac surgery. Thus, this polymorphism can actually not help to identify high risk groups in the heterogeneous population of individuals who undergo cardiac surgery with CPB.

## Background

Nitric oxide (NO) is an endothelium-derived relaxing factor (EDRF) which represents one of the most relevant molecules involved in biological systems. NO is synthesized by NO Synthase (NOS) from L-arginine. There are three types of NOS: inducible NOS (*iNOS*), neural NOS (*nNOS*), and endothelial NOS (*eNOS*) [1]. The human *eNOS* gene is located on the chromosome No. 7 (7q35-36) which consists of 26 exons with an entire length of 21 kb and is constitutively expressed in vascular endothelial cells. *eNOS* is the key enzyme responsible for basal vascular production of NO [2]. In addition to influencing relaxation of vascular smooth muscle cells, endothelium-derived NO inhibits platelets [3] and leukocytes [4] adhesion to vascular endothelium, inhibits vascular smooth cell migration and proliferation [5], and limits the oxidation of atherogenic low-density lipoprotein [6].

Furthermore, it has been shown that the production of NO is significantly increased during and after cardiopulmonary bypass (CPB) [7]. The systemic endotoxemia that occurs with the establishment of CPB is a potent stimulus for the release of proinflammatory cytokines which induce iNOS expression and subsequent NO release [8]. The increased release of NO due to expression of *iNOS* may not only contribute to the reduced activity of *eNOS*, but may also lead to increased vascular permeability and other manifestations of systemic inflammation after CPB [9,10]. Therefore, changes in *eNOS* activity and NO bioavailability after cardiac surgery with CPB may lead to vasomotor abnormalities with impaired regulation of myocardial perfusion, altered peripheral vascular resistance, and vascular permeability with endorgan edema.

Several polymorphisms in the gene encoding *eNOS* influence the production and functional activity of the enzyme. A substitution of guanine to thymine at nucleotide 894 in exon 7 of the *eNOS* gene (894G- > T) which leads to an amino acid change from Glu to Asp at codon 298 (also called Glu298Asp) was shown to reduce basal NO production [11]. Functional consequences suggest that 894 T SNP are associated with an up to 80% decreased *eNOS* activity and an increased susceptibility to cleavage of the *eNOS* protein of the T-genotypes, both resulting in decreased NO generation [12,13]. Some studies have shown that T-allele carriers have an increased risk of hypertension [14], coronary spasm [15], myocardial infarction [16], and coronary artery disease [17]. A recent clinical study indicates that patients who are homozygous for the T-allele, have an enhanced responsiveness to α_1_- adrenergic stimulation during cardiac surgery with CPB [18]. Based on these pathophysiological backgrounds, we conducted a prospective study to determine the influence of the *eNOS* G894T on early and late outcome after cardiac surgery with CPB. Early morbidity and long-term mortality after cardiac surgery with CPB were our key points of interest in this study.

## Methods

### Participants

After approval by the local ethics committee (University of Göttingen, Germany), data of 500 adult Caucasian patients who underwent cardiac surgery with CPB were analysed. Patients with known neoplasms were excluded from this observation. A written informed consent was obtained from each participant included in this prospective study. A part of the present patient population was used for several genetic investigations [19-21].

Genomic DNA was extracted from patient’s EDTA blood samples collected before surgery. After extracting the DNA, genotyping was performed and patients were divided in three groups according to their genotype (GG, GT, and TT) (Table 1).

**Table 1:** Endothelial 894 G/T NO-Synthase genotypes and allelic frequencies of patients

**894G/T**	**GG**	**GT**	**TT**	**G-allele**	**T-allele**	**HWE p-value**
n = 500	251 (50.2%)	210 (42%)	39 (7.8%)	712 (71%)	288 (29%)	0.86

### Definitions

The preoperative patient characteristics included age, gender, BMI (body mass index), smoking habits, hypertension, history of diabetes, renal disorder, hypercholesterolemia, positive family history of cardiovascular disorders, left ventricular ejection fraction, peripheral vascular disease, history of neurocerebral events, pulmonary hypertension, and chronic obstructive pulmonary disease. Additionally, preoperative medications, urgency of surgery, concomitant procedures, as well as additive Euroscore, Acute Physiology and Chronic Health Evaluation Score (APACHE II), and Simplified Acute Physiology Score (SAPS II) were recorded (Table 2).

**Table 2:** Baseline characteristics

**Variable**	**GG**	**GT**	**TT**	**p value**
	**(n = 251)**	**(n = 210)**	**(n = 39)**	
Age (years)	68 ± 10	67 ± 11	68 ± 9	0.97
Male/female	163/88	134/76	26/13	0.93
***Risk factors***				
Body mass index (kg/m^2^)	28 ± 4.6	27. ± 4.8	27 ± 4.5	0.18
Smoking	85	66	16	0.49
Hypertension	184	144	34	0.05
Hypercholesterolemia	125	95	21	0.48
Diabetes mellitus	78	63	13	0.91
Positive family history	40	31	8	0.66
Ejection fraction (%)	53 ± 14	52 ± 15	62 ± 10	0.07
Renal disorder	29	33	6	0.41
Peripheral disease	17	12	6	0.09
Neurocerebral events	36	26	4	0.70
Pulmonary hypertension	26	11	3	0.13
COPD	18	18	4	0.74
Dialysis	1	2	0	0.66
***Preoperative medications***				
ß-blockers	158	129	29	0.31
ACE inhibitors	135	103	25	0.19
Oral nitrates	55	33	5	0.15
Antiarrhythmics	11	7	1	0.77
Diuretics	106	80	13	0.46
Antilipid agents	119	94	25	0.08
Antidiabetics	51	35	8	0.58
Antihypertensive agents	54	49	8	0.87
Bronchodilators	6	8	1	0.66
Anticoagulation	163	145	29	0.40
Urgency of surgery				
Elective (n = 396)	202	161	33	0.41
Urgent (n = 41)	26	13	2	0.21
Emergency (n = 63)	23	36	4	0.03
***Associated surgical procedures***				
CABG (n = 251)	118	111	22	0.26
Valve (n = 107)	58	44	5	0.32
Combined procedures (n = 101)	55	36	10	0.36
Other procedures (n = 41)	20	19	2	0.78
Euroscore additive	5 ± 4	6 ± 4	5 ± 3	0.19

COPD: chronic obstructive pulmonary disease, ACE: angiotensin-converting enzyme, CABG: Coronary artery bypass grafting.

Perioperative patient characteristics, such as intraoperative cross-clamp and cardiopulmonary bypass time, oxygenation index (PaO2/FiO2), positive end-expiratory pressure (PEEP), partial pressure of carbon dioxide (PCO2), arterial pH, compliance, presence of infiltrates and lung injury score were prospectively evaluated intra- and over 24 hours postoperatively. Hemodynamic measurements comprised heart rate (HR), mean arterial pressure (MAP), central venous pressure (CVP), and mean pulmonary artery pressure (PAP), pulmonary capillary wedge pressure (PCWP). Cardiac index (CI), systemic vascular resistance (SVRI) and pulmonary vascular resistance (PVRI) indices were calculated using routine formulas. Extended hemodynamic measurements were done in cases of poor condition and inotropic dependency. Additionally, catecholamine support, administration of amiodarone, cortisone, nitroglycerin, and vasopressin were recorded. All perioperative variables are summarized in Table 3.

**Table 3:** Perioperative data

**Variable**	**GG**	**GT**	**TT**	***p *** **value**
	**(n = 251)**	**(n = 210)**	**(n = 31)**	
***Pulmonary function***				
Pa_O2_/Fi_O2_	262 ± 108	259 ± 95	244 ± 9	0.80
PEEP (mbar)	7 ± 2	7 ± 2	8 ± 3	0.50
PCO2 (mmHg)	40 ± 5	40 ± 4	40 ± 4	0.67
pH arterial	7.39 ± 0.05	7.39 ± 0.05	7.39 ± 0.05	0.50
Compliance (ml/mbar)	47 ± 21	45 ± 18	50 ± 31	0.59
Infiltrates (Quadrants)	1.24 ± 1	1.34 ± 1	1.31 ± 1	0.46
Lung injury score	1.28 ± 1	1.34 ± 1	1.33 ± 1	0.55
***Scores***				
APACHE II Score	14 ± 6	16 ± 7	16 ± 6	0.06
SAPS II Score	25 ± 7	25 ± 8	25 ± 6	0.51
***Hemodynamic***				
Heart rate (bpm)	81 ± 12	82 ± 14	85 ± 15	0.46
MAP (mmHg)	80 ± 8	80 ± 9	82 ± 8	0.25
CVP (mmHg)	11 ± 4	11 ± 3	11 ± 3	0.76
PCWP(mmHg)	16 ± 4	14 ± 5	15 ± 2	0.07
PAP (mmHg)	25 ± 5	26 ± 9	24 ± 9	0.83
CI (l/min/m^2^)	3.2 ± 3.2	2.8 ± 0.8	2.7 ± 0.4	0.99
SVRI (dyn∙s-1∙m^2^∙cm-5)	958 ± 394	987 ± 403	861 ± 213	0.83
PVRI (dyn∙s-1∙m^2^∙cm-5)	202 ± 177	219 ± 155	189 ± 81	0.36
***Inotropes***				
Epinephrine (mg/d)	9.6 ± 129	3.4 ± 15	0.5 ± 1.4	0.24
Norepinephrine (mg/d)	1.4 ± 6.0	4.6 ± 19.3	0.2 ± 0.66	0.03
Enoximone (mg/d)	17 ± 107	29 ± 200	0.2 ± 1.28	0.57
Dobutamine (mg/d)	10 ± 50	11 ± 63	21 ± 58	0.29
***Other agents***				
NTG (mg/d)	10 ± 25	12 ± 32	13 ± 20	0.24
Amiodarone (mg/d)	28 ± 159	105 ± 361	62 ± 10	0.01
Cortisone (mg/d)	44 ± 367	37 ± 227	9 ± 58	0.12
Vasopressin (iU/d)	0.16 ± 2.5	0.19 ± 1.89	0	0.40
***Operative characteristics***				
Cross-clamp time (min)	93 ± 37	93 ± 39	105 ± 42	0.27
Cardiopulmonary bypass time (min)	143 ± 75	141 ± 58	162 ± 69	0.15

PEEP: positive end-expiratory pressure, APACHE II Score: Acute Physiology and Chronic Health Evaluation Score, SAPS II Score: Simplified Acute Physiology Score, HR: Heart rate, MAP: mean arterial pressure, CVP: central venous pressure, PCWP: pulmonary capillary wedge pressure, PAP: mean pulmonary artery pressure, CI: cardiac index, SVRI: systemic vascular resistance, PVRI: pulmonary vascular resistance, NTG: nitroglycerin.

Postoperative details included transfusion and clotting factor requirement, intraaortic ballon pump (IABP) usage, need for extracorporeal membrane oxygenation (ECMO) support, length of hospital and intensive care unit (ICU) stay, and in-hospital mortality (Table 4).

**Table 4:** Postoperative course

**Variable**	**GG**	**GT**	**TT**	***p *** **value**
	**(n = 251)**	**(n = 210)**	**(n = 39)**	
Red blood cells transfused (ml/d)	215 ± 554	298 ± 684	267 ± 398	0.05
Fresh Frozen Plasma (ml/d)	64 ± 387	97 ± 702	33 ± 127	0.66
Prothrombin complex concentrates (iU/d)	6 ± 94	17 ± 184	0	0.66
IABP	15	21	1	0.13
ECMO	0	1	0	0.50
Length of ICU stay (d)	6 ± 12	10 ± 17	5 ± 6	0.001
Hospitality stay (d)	25 ± 20	27 ± 22	22 ± 11	0.91
***Early Mortality (n = 41)***				
Overall (8.2%) (%) n = 500	18	20	3	0.65
Elective cases (%) n = 396	11	14	1	0.32
Urgent cases (%) n = 41	2	3	1	0.15
Emergency cases (%) n = 63	5	3	1	0.29
***Long-term Mortality (n = 125)***				
Overall (25%) (%) n = 500	64	50	11	0.88
Age at surgery (years)	72 ± 8	72 ± 7	73 ± 4	0.67
Survival after surgery (days)	500 ± 493	497 ± 588	599 ± 578	0.71
Age at death (years)	74 ± 8	73 ± 7	75 ± 4	0.64

IABP: intraaortic ballon pump, ECMO: extracorporeal membrane oxygenation, ICU: intensive care unit.

Non-elective operation was defined as the necessity of surgery within the same week of referral (urgent case) or the necessity to take the patient to theatre out of normal working hours and before the next morning’s operating list (emergency cases). In-hospital mortality was defined as intraoperative death or mortality during the same hospitalization. A five-year follow-up for all initially survived patients was performed with the view to assessing the long-term mortality.

### Genotyping

Genomic DNA was extracted from peripheral blood leukocytes using a commercially available kit and was performed using polymerase chain reaction amplification according to previously described protocols [22]. Patients were divided into the following three groups according to their genotypes: GG, GT, and TT. The clinical data were blinded to the laboratory personal who performed genetic analysis.

### Statistical analysis

Prior to commencement of this study, a power analysis was done. The calculated appropriate sample size needed to attain a given power of 80% and to achieve a significance of p < 0.05 was 500 patients. All statistical analyses were computed by the commercially available software (SPSS for Windows, Version 13.0, SPSS Inc. Chicago, USA). Continuous variables are presented as the mean ± SD, and categorical variables are presented as absolute numbers or percentages. Data were evaluated for normality before statistical analysis. Groups were compared using analysis of variance for normally distributed data or by the nonparametric Kruskal–Wallis test for non-normally distributed variables. The Scheffe’ post hoc test was used as appropriate.

Contingency table methods, including the Chi-square test and the Fisher exact test, were used to analyse categorical data. Allele frequencies in the study population were counted and compared to the expected distribution in normal population by Hardy-Weinberg’s equilibrium and checked by χ^2^-test. Statistical difference was considered at p < 0.05.

The Kaplan–Meier method was used for survival analysis and the log rank test was performed for the comparison among the groups in terms of mortality.

## Results

### Baseline characteristics

The G894T polymorphism of the *eNOS* gene was determined, and 3 groups were defined according to the genotype (GG, GT, and TT). Of the studied subjects 251 patients were homozygous G-allele carrier (GG), 210 patients were heterozygous for the G894T polymorphism (GT) and 39 patients were homozygous (TT) for the transversion in this gene. According to this, we found an allele frequency of 0.71 for G-allele and 0.29 for T-allele in studied subjects. Genotype distribution of the *eNOS* G894T polymorphism was consistent with the *Hardy-Weinberg equilibrium*. The genotype frequencies and allelic of the polymorphism in this study closely matched those published in previous studies [18,20] and are summarized in Table 1. There were no significant differences in baseline characteristics (Table 2), except for urgency of surgery. In the GT group more patients underwent emergency surgery (p = 0.03).

### Perioperative characteristics

No differences were observed in the statistical analysis with regard to PaO2/FiO2, PCO2, arterial pH, PEEP, lung injury score, APACHE II score, and SAPS II score. Hemodynamic measurements performed over 24 hours postoperatively revealed no significant differences among the three groups concerning HR, MAP, CVP, PCWP, PAP, CI, SVRI and PVRI. There was no difference in consumption of epinephrine, enoximone, dobutamine, nitroglycerine (NTG), vasopressin, and cortisone. However, the consumption of norepinephrine (p = 0.03) and amiodarone (p = 0.01) was significantly higher in patients who were heterozygous for the *eNOS* 894G/T polymorphism.

Furthermore, no difference was observed with regard to CPB time and aortic cross clamp time. All results are summarized in Table 3.

### Postoperative course

The postoperative morbidity and in-hospital mortality are presented in Table 4. There was no relation between red blood cells transfusion, administration of fresh frozen plasma or prothrombin complex concentrates, and genotype. IABP and ECMO usage was comparable in all groups. The mean postoperative hospital length of stay was also comparable among the genotype carriers. However, the mean ICU stay showed statistical differences among the groups. Patients who were heterozygous for the *eNOS* G894T polymorphism had a prolonged stay at the ICU (p = 0.001).

The overall in-hospital mortality rate was 8.2% for all patients included in this study (41 of 500) and did not reach statistical significance comparing the three groups (GG: n = 18; GT: n = 20; TT: n = 3) (*p* = 0.65). Furthermore, the in-hospital mortality was quite uniform across urgent and emergency cases and no significant correlation among genotypes was observed.

### Long-term mortality

**Figure 1:**
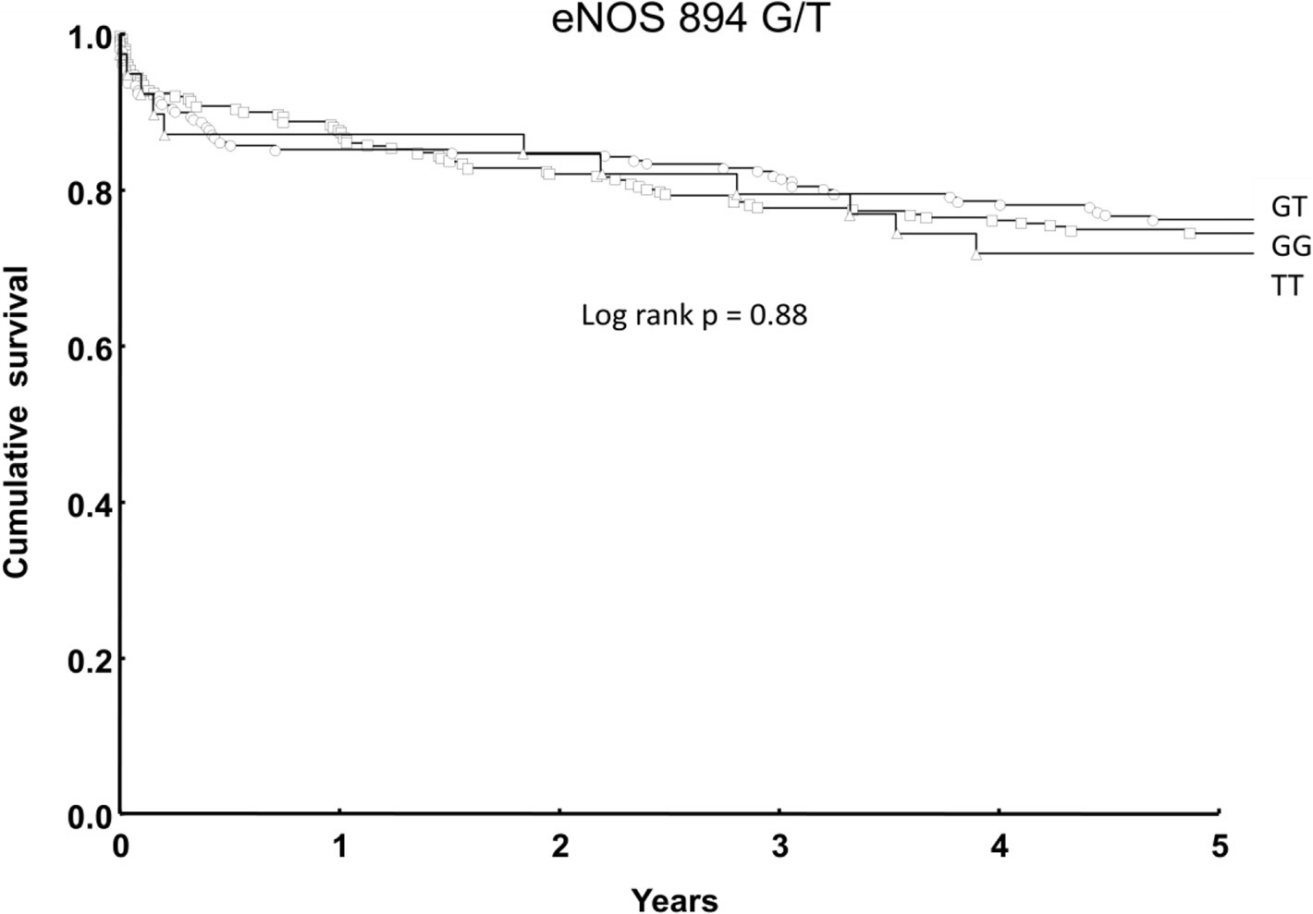
Cumulative survival (overall mortality after 5 years was 25%).

Long-term follow-up of initial survivors was available in all cases (100%) and median follow-up time was 5 years. During this period, 12 patients died from cardiac related complications, 10 patients died from multi organ failure, 8 died from cerebrovascular accidents, 5 patients had a cancer related death, and in the remaining 45 patients the cause of death was unknown (n = 84). The overall mortality was 25% (n = 125) and there was no significant difference in the statistical analysis with regard to genotypes distribution (GG: n = 66, GT: n = 50, TT: n = 11, log rank test p = 0.88) (Figure 1).

## Discussion

This study examined the association of *eNOS* G894T polymorphism with in-hospital mortality and morbidity after cardiac surgery with CPB. The investigated *eNOS* gene polymorphism revealed no different risk for the detected homozygous TT genotype. Furthermore, homozygous TT carriers did not contribute to a higher prevalence of postoperative in-hospital mortality after cardiac surgery with CPB. Our genotype frequencies for *eNOS* G894T polymorphism were in accordance with previously reported results in other European populations [18,23].

NO plays a crucial role in regulating a wide spectrum of functions in the cardiovascular system, including vasorelaxation, vascular smooth muscle cell migration and proliferation, as well as platelet aggregation. Previous studies provide evidence that defects of endothelial NO function cause endothelial dysfunction [24,25]. Moreover, open heart surgery with CBP remains associated with systemic inflammation and suboptimal outcome in many individuals. Systemic hypotension, myocardial failure, increased vascular permeability and consequent dysfunction of endorgans are manifested by inflammatory changes and changes of *eNOS* activity [26,27]. The increased release of inflammatory substances may potentially lead to changes in vasomotor regulation, endothelial integrity and vascular permeability that may compromise the recovery of patients undergoing cardiac surgery with CPB [23].

Several genetic polymorphisms of the *eNOS* gene have been reported as “susceptibility genes” in a number of cardiovascular diseases. It has been shown, that the *eNOS* G894T polymorphism is associated with reduced basal NO [11]. In addition, this polymorphism was associated with hypertension [14], coronary spasm [15] increased risk for myocardial infarction [16], and coronary artery disease [17]. In a previous study, the *eNOS* G894T polymorphism was reported to be associated with an enhanced vascular responsiveness to phenylephrine and influenced systemic hemodynamics in patients undergoing surgery with CPB [18], thus suggesting a difference in vascular reactivity among genotype carriers. Our results are partially in accordance with those findings. In the present study extended systemic hemodynamic analyses were performed during the perioperative period in cases of poor condition. An interaction between the *eNOS* G894T polymorphism and perioperative hemodynamics was not observed within the first 24 hours. Otherwise, the differences in norepinephrine support (p = 0.03) and administration of amiodarone (p = 0.03) reached statistical significance for patients who were heterozygous for the eNOS 894G/T polymorphism. Although, T allele carriers did not show a decrease of SVRI and PVRI after cardiac surgery with CPB, there might be an influence on the postoperative hemodynamics. Furthermore, in our study, we detected no relationship between the T-allele and increased aortic cross-clamp time and CPB time. Therefore, we can exclude the possibility that this may have influenced the results of the present study. It might have been possible, that SVRI and PVRI in T allele carriers could have achieved statistical difference, if administration of catecholamine had been declined. Our observation may be explainable, because in cases of impaired hemodynamics, patients were immediately treated with drugs to avoid hemodynamic complications. Another reason why we failed to show statistical differences in hemodynamics could be that we did not use a dominant or additive model of inheritance. Philip et al. combined all T-allele carriers (GT + TT) to a single group (dominant model of inheritance), because the sample size was less [18]. Liakopoulos et al. did not detect significant associations between this polymorphism and changes in hemodynamics after cardiac surgery with CPB [22] and have also used the dominant model of inheritance. Our observation is more powerful compared to previous research, as the number of subjects used in our study is larger than that in two studies previously reported.

Relatively small sample size can be a significant contributory factor for the failure to detect and replicate associations across studies. Interestingly, Ruel et al. demonstrated that altered release of NO after cardiac surgery with CPB may lead to reduced peripheral and pulmonary resistance, and abnormal vascular permeability. Furthermore, these abnormalities may lead to organ dysfunction, such as organ edema, impaired regulation of myocardial perfusion and can contribute to myocardial and endothelial dysfunction [28]. Thus, this observed vasomotor dysfunction might be induced by the *eNOS* G894T polymorphism and/or CPB leading to an increased susceptibility to cleavage of the *eNOS* protein of the T genotypes.

The investigations on the influence of the *eNOS* G894T polymorphism on early clinical outcome in patients undergoing cardiac surgery with CPB was the primary endpoint of the present study.

The major finding is that the *eNOS* G894T polymorphism did not show an interaction with the clinical outcome in patients undergoing cardiac surgery. The operative morbidity and mortality of patients underwent cardiac surgery with CPB was low. There was no conduction disturbance. The overall in-hospital mortality rates observed in the present study are acceptable and were comparable to 30-day mortality in cardiac surgery [29]. However, in our study GT allele carriers had a prolonged ICU stay (p = 0.001). This finding is not surprising, because significant more GT allele carriers underwent an emergency operation (p = 0.03). Urgent and/or emergent surgery has long been known to be a risk factor for prolonged ICU stay [30]. Moreover, in the long-term follow-up which was 100% complete, there was no influence of the *eNOS* G894T polymorphism on mortality. We observed an overall mortality of 25% after five years which is acceptable for heterogeneous elderly cardiac surgical patients including emergency procedures and combined procedures.

It is important to note that this study has several limitations. Although our patients were distributed according to *Hardy-Weinberg equilibrium*, our study is limited by the relatively small number of patients in the TT group, which may have affected the clinical outcome. Because the study population was selected from patients undergoing cardiac surgery, the sample size was smaller than in gene polymorphism studies with non-surgical patients suffering from cardiovascular disorders [16,17]. Another limitation is a short perioperative observation period only lasting for 24 hours postoperatively. The influence of the *eNOS* G894T polymorphism might have been higher and the sensitivity and specificity for analysis of data would have been more appropriate if the recording time had been longer. However, to our knowledge, this is the first investigation on the *eNOS* G894T polymorphism regarding its clinical impact on early and late mortality in cardiac surgery patients.

## Conclusions

In conclusion, the results of the present study demonstrate that homozygous T-allele carriers do not have a significantly increased risk for in-hospital or long-term mortality after cardiac surgery with CPB. Thus, the polymorphism can actually not help to identify high risk groups in the heterogeneous population of individuals who undergo cardiac surgery with CPB.

## Competing interests

The authors declare that they have no competing interests.

## Authors’ contributions

JH and AFP conceived the study, participated in its designed and coordination. DS, CL, and CB were involved in data acquisition. OM, IB, CHW, and AM were involved in the medical treatment of the patients on intensive care unit. HS and AS helped to draft the manuscript. MQ and FAS gave important comments. All authors read and approved the final manuscript.
